# Long‐term stability of microbiome diversity and composition in fecal samples stored in eNAT medium

**DOI:** 10.1002/mbo3.1046

**Published:** 2020-05-10

**Authors:** Rebecca R. Young, Kirsten Jenkins, Felix Araujo‐Perez, Patrick C. Seed, Matthew S. Kelly

**Affiliations:** ^1^ Division of Pediatric Infectious Diseases Duke University Durham NC USA; ^2^ Department of Pediatrics Feinberg School of Medicine Northwestern University Chicago IL USA

**Keywords:** 16S rRNA sequencing, bacterial inactivation, biological sample shipping, stabilization media

## Abstract

Fecal samples collected for microbiome analyses are typically frozen to avoid postcollection changes in microbial composition. eNAT is a guanidine thiocyanate‐based medium that stabilizes microbial DNA and allows safe specimen handling and shipping by inactivating microorganisms. We collected fecal samples (*n* = 50) from children undergoing hematopoietic stem cell transplantation. We divided samples into three aliquots: (a) stored in RNAlater and immediately transferred to −80°C; (b) stored in eNAT medium and immediately transferred to −80°C; and (c) stored in eNAT medium at ambient temperature (~20°C) for 30 days prior to transfer to −80°C. Mean (standard deviation) Shannon diversity and Chao1 indices in sample aliquots were 2.05 (0.62) and 23.8 (16.6), respectively. Comparing samples frozen immediately in RNAlater to samples frozen immediately in eNAT, there were no differences in Shannon diversity (*p* = .51), Chao1 richness (*p* = .66), and overall microbiome composition (*p* = .99). Comparing eNAT samples frozen immediately to samples stored at ambient temperature, we identified no differences in Shannon diversity (*p* = .65), Chao1 richness (*p* = .87), and overall microbiome composition (*p* = .99). Storage of fecal samples in eNAT at ambient temperature for 30 days did not alter microbiome richness, diversity, or composition. eNAT may be a useful medium for fecal microbiome studies, particularly when cold chain storage is unavailable.

## Graphical abstract

eNAT® medium is a guanidine thiocyanate‐based medium that stabilizes microbial DNA and allows safe specimen handling and shipping by inactivating live microorganisms. In comparing eNAT samples frozen immediately to eNAT samples stored at ambient temperature, we identified no detectable differences in Shannon diversity (*p* = .65), Chao1 richness (*p* = .87), overall microbiome composition (*p* > .99), or the relative abundances of highly abundant genera. eNAT may be a useful medium for fecal microbiome studies, particularly when cold chain storage is unavailable.

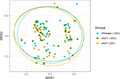

**Abbreviations**
ANOVA: analysis of variance
PCR: polymerase chain reaction
PERMANOVA: permutational multivariate analysis of variance using distance matrices

## 1 INTRODUCTION

Gut microbial communities are fundamental to human health, with a growing body of literature supporting a role for the gut microbiome in immune development and function (Gong et al., 2019), metabolism (Henderickx, Zwittink, van Lingen, Knol, & Belzer, 2019), early childhood growth (Blanton et al., 2016), and protection from infection (Yang & Duan, 2018). With the recent development of high‐throughput sequencing technologies, the gut microbiome is now most commonly studied through the isolation of DNA from fecal samples. Fecal samples collected for this purpose are typically rapidly frozen to −20°C (or colder) to prevent microbial growth that can occur after collection (Roesch et al., 2009). An alternative approach to minimizing this postcollection bias is to collect samples into a medium that inactivates bacteria and stabilizes DNA (Choo, Leong, & Rogers, 2015). Use of these stabilization media may be particularly useful for microbiome studies that involve the collection of clinical samples outside of healthcare settings. For instance, these media enable fecal samples collected in the home environment to be stored at ambient temperature, obviating the need for storage in a refrigerator or freezer alongside food items. Additionally, stabilization media may be essential to microbiome studies conducted in regions of the world where there is no cold chain storage and where quickly transporting samples to a freezer is not possible or would be cost‐prohibitive (Choo et al., 2015). Finally, use of stabilization media and storage at ambient temperature could minimize DNA degradation associated with freeze–thaw cycles during sample transport or processing (Song et al., 2016).

Tris‐EDTA buffer and 70%–99% ethanol have historically been the most common stabilization media used in microbiome research, but there are several challenges to the use of these media (Choo et al., 2015; Vandeputte, Tito, Vanleeuwen, Falony, & Raes, 2017). First, buffers containing EDTA may not optimally preserve the microbial composition of fecal samples (Reidmiller et al., 2006). In one study, samples stored in Tris‐EDTA at ambient temperature had lower abundances of *Bifidobacterium* and *Anaerostipes* and higher abundances of *Bacteroides* and *Proteobacteria* than samples stored in Tris‐EDTA buffer and frozen immediately to −80°C (Choo et al., 2015). Similarly, shifts in microbiome diversity and composition have been reported with the storage of fecal samples in 70% ethanol at ambient temperature (Song et al., 2016). In contrast, 95% ethanol effectively preserves gut microbial diversity and composition at ambient temperature for at least 8 weeks (Song et al., 2016), but this solution is highly flammable and expensive to transport (Bentley, 2007; Nagy, 2010).

In this study, we sought to evaluate the potential utility of eNAT^®^ (Copan Italia,) as a stabilization medium for microbiome research. eNAT is a commercially available medium developed for the preservation and stabilization of DNA and RNA in clinical specimens used for nucleic acid tests. This medium contains a protein denaturant (guanidine thiocyanate) that completely inactivates microorganisms, does not require special shipping precautions, and is marketed as being able to stabilize DNA at room temperature for up to 4 weeks (Chomczynski & Sacchi, 2006). We compared microbiome diversity and composition of fecal samples stored in a standard microbiome medium (RNAlater solution and immediately frozen) to matched fecal samples stored in eNAT and immediately frozen. Additionally, we evaluated the stability of the gut microbiome in fecal samples stored in eNAT at ambient temperature for 30 ± 2 days.

## 2 METHODS

### 2.1 Sample collection

This study made use of fecal samples collected between January 2018 and June 2018 through a prospective cohort study of children and adolescents (<18 years of age) undergoing hematopoietic stem cell transplantation at Duke University. The primary objective of this study is to evaluate the utility of serial sampling of the gut microbiome for the prediction of infections after stem cell transplantation. Fecal samples were collected into 50‐mL sterile collection tubes containing a small amount (~2.5˗5.0 ml) of RNAlater solution and placed immediately into a 4°C refrigerator. RNAlater solution inhibits bacterial growth but leaves bacterial cells intact and viable (Life Technologies, 2011).

### 2.2 Sample processing

Fecal samples were collected in the hospital and transported to the laboratory daily (Monday–Friday) by research team members for processing and storage. Samples were vortexed in the collection tube, and aliquots of the resulting stool slurry were distributed as follows: (a) 0.5 ml was placed into a cryovial containing 1.0 ml of RNAlater solution and immediately transferred to a −80°C freezer; (b) 0.5 ml was placed into a cryovial containing 1.0 ml of eNAT medium and immediately transferred to a −80°C freezer; and (c) 0.5 ml was placed into a cryovial containing 1.0 ml of eNAT medium, allowed to sit in the laboratory at ambient temperature (~20°C) for 30 ± 2 days, and then transferred to a −80°C freezer.

### 2.3 DNA extraction and bioinformatics

Total genomic DNA was extracted using an established protocol involving mechanical and enzymatic lysis (LaTuga et al., 2011). PCR was used to amplify the variable V4 region of the bacterial 16S ribosomal RNA gene using an Illumina MiSeq instrument (Illumina Inc.). Sequencing reads were split, quality‐trimmed, and demultiplexed with the use of QIIME tools (Caporaso et al., 2010). A DADA2 pipeline was used to remove chimeric variants and to identify amplicon sequence variants (Callahan et al., 2016). Taxonomic assignments were made based on alignment with the Greengenes database (Larsen et al., 2006). Samples with less than 1,000 sequencing reads were pruned. In addition, amplicon sequence variants with a mean relative abundance of less than 5 × 10^–6^ were filtered.

### 2.4 Statistical analyses

The Shannon diversity and Chao1 index were calculated for each sample (McMurdie & Holmes, 2013). We performed a mixed‐model ANOVA model where the independent variable of interest was the sample preparation method, and the outcome was either the Shannon diversity index or the Chao1 index (Moser, 2004). We applied a natural log transformation of the Chao1 index before ANOVA because this statistic was not normally distributed in study samples. To account for children having repeated samples and the fecal samples being split into aliquots, we included a random effect of a participant, where the sample was nested within the participant. A Bland–Altman plot was used to describe the agreement between the Shannon diversity index, and separately the Chao1 index, with pairwise sample preparation methods (Altman & Bland, July, 1983). The Bray–Curtis nonmetric multidimensional scaling matrix was calculated. We used permutational multivariate analysis of variance using distance matrices (PERMANOVA) to determine whether there were global differences in fecal microbiota composition according to the sample preparation method (Oksanen et al., 2019). We implemented zero‐inflated Gaussian mixture models using the R package metagenomeSeq to identify genera that were differentially abundant by preparation method (Paulson, Pop, & Bravo, 2013) All analyses were conducted by a statistician blinded to the sample preparation method. The analyst was blinded to sample preparation method during analysis.

## 3 RESULTS

**Figure 1:**
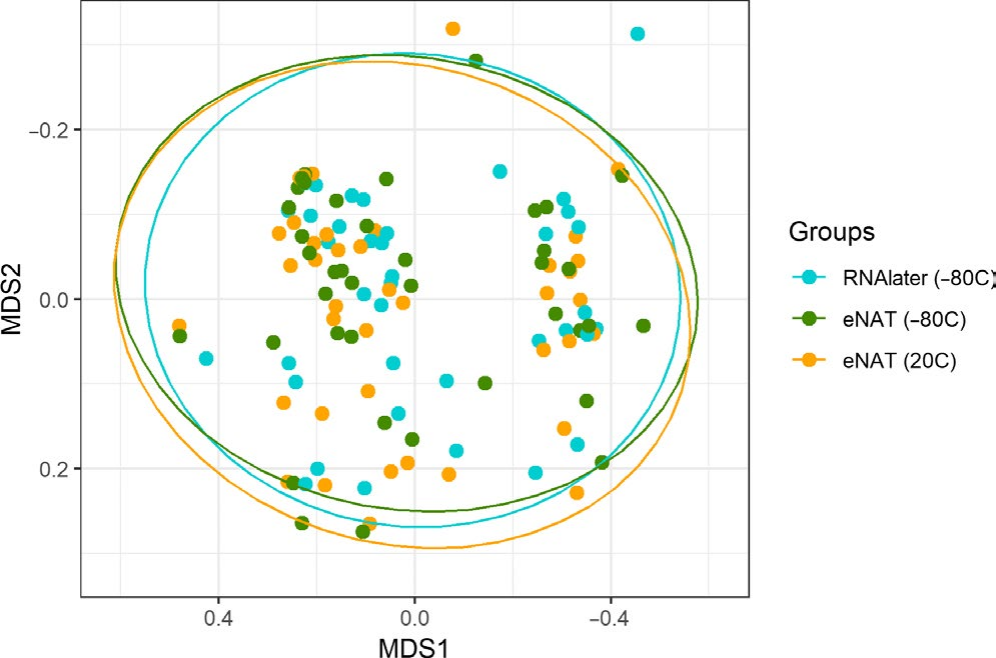
Similar microbiome composition of matched fecal sample aliquots prepared in eNAT (−80°C), RNAlater (−80°C), and eNAT (20°C). The nonmetric multidimensional scaling distances of the Bray–Curtis matrix show no difference in global composition by sample preparation method (PERMANOVA; *p* = .99)

The 50 fecal samples included in this study were collected from 13 subjects. After pruning of low‐read samples (<1,000 reads), there were matched aliquots of RNAlater solution followed by immediate freezing to −80°C and eNAT followed by immediate freezing to −80°C for 39 fecal samples. Similarly, there were matched aliquots of eNAT followed by immediate freezing and eNAT with storage at ambient temperature for 39 fecal samples. A total of 2,145,517 high‐quality 16S ribosomal RNA sequences (mean of 17,876 sequences per sample aliquot) were obtained from the 120 sample aliquots included in this study. Rarefaction curves were constructed to ensure complete coverage of the bacterial diversity at the given sequencing depth (Figure A1). The mean (*SD*) Shannon diversity and Chao1 indices in sample aliquots were 2.05 (0.62) and 23.8 (16.6), respectively. Sequences were assigned to 526 amplicon sequence variants (after filtering) representing 117 genera from 10 phyla. Table 1 shows the relative abundances of the 20 most abundant bacterial genera identified in sample aliquots included in these analyses. The most highly abundant bacterial genera were *Bacteroides* (34.6%), *Enterococcus* (17.0%), *Parabacteroides* (7.3%), and *Clostridium* (6.2%). Figure 1 shows nonmetric multidimensional scaling matrices of fecal microbiota communities by sample preparation method. We observed no difference in the overall microbiome composition of samples prepared in RNAlater with immediate freezing, samples prepared in eNAT with immediate freezing, and samples prepared in eNAT and stored at 20°C for 30 ± 2 days (PERMANOVA, *p* > .99).

**Table 1:** Mean and maximum relative abundance of the 20 most highly abundant bacterial genera in fecal samples

Genus	Mean relative abundance	Maximum relative abundance
*Bacteroides*	34.6%	99.6%
*Enterococcus*	17.0%	93.0%
*Parabacteroides*	7.3%	73.9%
*Clostridium* (Clostridiaceae)	6.2%	69.6%
*Faecalibacterium*	4.9%	28.0%
*Lactobacillus*	4.1%	82.7%
*Prevotella*	3.8%	62.8%
*Oscillospira*	3.4%	30.9%
*Ruminococcus* (Lachnospiraceae)	2.5%	5.0%
*Bifidobacterium*	1.6%	31.5%
*Streptococcus*	1.5%	19.2%
*Blautia*	1.4%	21.5%
*Ruminococcus* (Ruminococcaceae)	1.3%	6.1%
*Alistipes*	1.3%	17.0%
*Lachnospiraceae*	1.3%	5.1%
*Sutterella*	1.2%	16.4%
*Campylobacter*	0.9%	53.2%
*Clostridium* (Erysipelotrichaceae)	0.9%	11.1%
*Roseburia*	0.4%	5.4%
*Pediococcus*	0.3%	15.0%

### 3.1 Gut microbial diversity and composition are not different in RNAlater solution and eNAT

**Figure 2:**
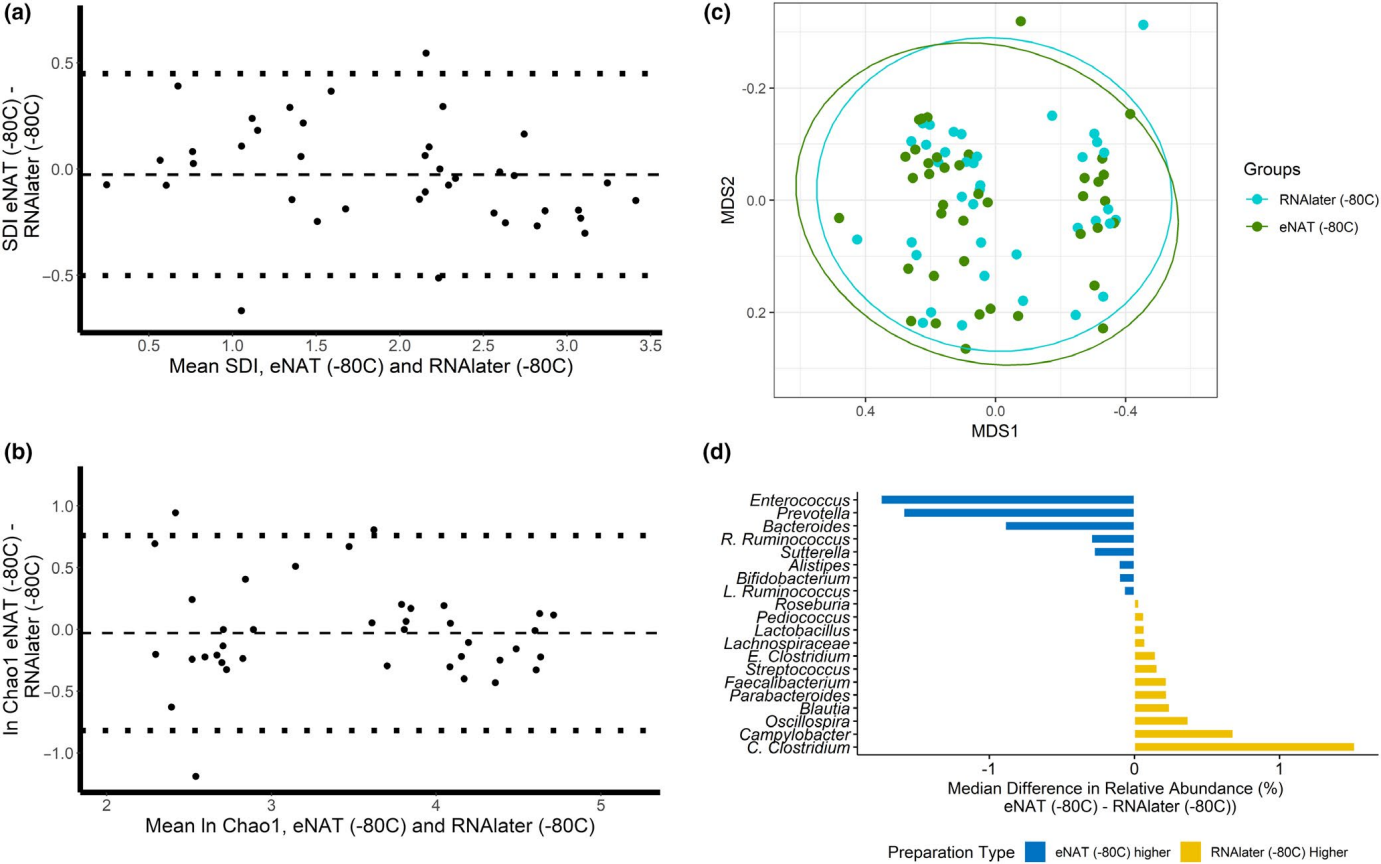
Matched fecal sample aliquots stored in eNAT and RNAlater and frozen immediately to −80°C do not have significantly different SDI, Chao1, global microbiome composition, and relative abundances of common bacterial genera. (a) A Bland–Altman plot of the Shannon diversity shows no proportional bias between sample preparation methods. Differences in the Shannon diversity index of matched sample aliquots are plotted on the *y*‐axis, and the mean of the Shannon diversity index of matched sample aliquots is plotted on the *x*‐axis; (b) A Bland–Altman plot of the natural log of the Chao1 index shows no proportional bias between sample preparation methods; (c) The nonmetric multidimensional scaling distances of the Bray–Curtis matrix show no difference in global microbiome composition by sample preparation method (PERMANOVA; *p* = .99); and (d) The median differences of relative abundances of the 20 most abundant bacterial genera are between 1.5% higher in RNAlater and 1.6% higher in eNAT

Mean (95% confidence interval [CI]) Shannon diversity and geometric mean (95% CI) Chao1 richness for sample aliquots placed into RNAlater solution with immediate freezing to −80°C were 1.94 (1.65, 2.23) and 34.4 (25.9, 45.7), respectively. Mean (95% CI) Shannon diversity and geometric mean (95% CI) Chao1 richness for samples aliquots stored in eNAT with immediate freezing were 1.92 (1.64, 2.20) and 33.4 (25.3, 44.0), respectively. There were no significant differences in the Shannon diversity index (*p* = .51) or the Chao1 index (*p* = .66) by the sample preparation method. Bland–Altman plots depicting differences in diversity measures of the gut microbiota in paired sample aliquots processed in RNAlater compared with eNAT are shown in Figure 2a,b. We found no evidence of proportional bias by sample preparation method across a broad range of values for Shannon diversity and Chao1 richness. We next sought to compare gut microbial composition in sample aliquots in RNAlater solution and sample aliquots in eNAT. Figure 2c displays the multidimensional position of fecal microbiota communities by sample preparation method. Global gut microbiota composition did not differ between sample aliquots prepared in RNAlater solution with immediate freezing and sample aliquots prepared in eNAT with immediate freezing (PERMANOVA; *p* > .99). Moreover, we found that the relative abundances of the 20 most abundant bacterial genera were not significantly different by the sample preparation method (Figure 2d). The median difference in relative abundance in paired sample aliquots was <2.5% for all 20 bacterial genera tested (Figure 2d). Among these 20 bacterial genera, there were no significant differences in abundance by sample preparation method (Table A2).

### 3.2 Stability of gut microbial diversity and composition in samples stored in eNAT at ambient temperature

**Figure 3:**
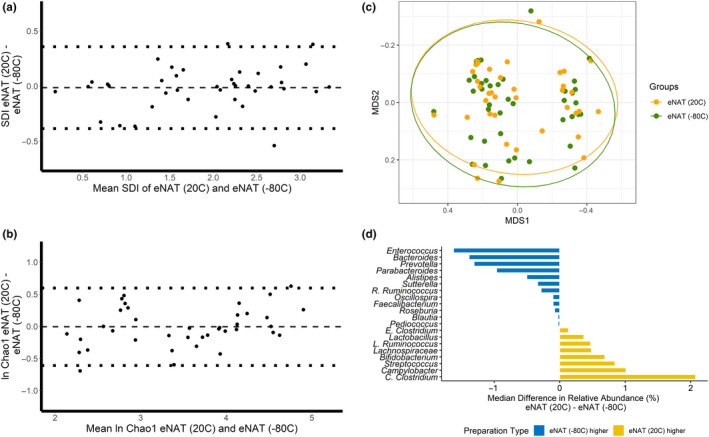
Matched fecal samples aliquots stored in eNAT and frozen immediately to −80°C and stored in eNAT at ambient temperature do not have significantly different SDI, Chao1, global composition, and genus relative abundance. (a) A Bland–Altman plot of the Shannon diversity shows no proportional bias between sample preparation methods; (b) A Bland–Altman plot of the natural log‐transformed Chao1 index shows no proportional bias by sample preparation method; (c) The nonmetric multidimensional scaling distances of the Bray–Curtis Matrix show no difference in global microbiome composition by sample preparation method (PERMANOVA; *p* = .99); and (d) The median differences of relative abundances of the 20 most abundant bacterial genera are between 1.6% higher in eNAT frozen immediately to −80°C and 2.1% higher in eNAT stored at ambient temperature

Mean (95% CI) Shannon diversity and the geometric mean (95% CI) Chao1 richness for samples aliquots stored in eNAT with immediate freezing to −80°C were 1.92 (1.60, 2.20) and 34.2 (25.3, 45.8), respectively. Mean (95% CI) Shannon diversity and geometric mean (95% CI) Chao1 richness for sample aliquots stored in eNAT at ambient temperature for 30 ± 2 days were 1.92 (1.66, 2.19) and 34.1 (25.4, 45.3), respectively. There were no significant differences in the Shannon diversity index (*p* = .65) or the Chao1 index (*p* = .91) by the sample preparation method. Bland–Altman plots depicting differences in diversity measures of the gut microbiota in paired sample aliquots processed in eNAT and immediately frozen to −80°C or stored at ambient temperature are shown in Figure 3a,b. We again found no evidence of proportional bias by sample preparation method across a broad range of values for Shannon diversity and Chao1 richness. Figure 3c displays the multidimensional position of fecal microbiota communities in sample aliquots placed in eNAT and immediately frozen to −80°C and sample aliquots placed in eNAT and stored at ambient temperature. Global gut microbiota composition did not differ by sample preparation method (PERMANOVA; *p* = .99). Moreover, we found that the relative abundances of the 20 most abundant bacterial genera were not significantly different by the sample preparation method (Figure 3d). The median difference in relative abundance in paired sample aliquots was 0.06% for all 20 bacterial genera tested (Figure 3d). There were no significant differences in the relative abundances of these bacterial genera by sample preparation method (Table A2).

## 4 DISCUSSION

This study demonstrates that the microbial diversity and composition of human fecal samples are not significantly different between storage in eNAT medium compared with RNAlater solution, a standard microbiome medium. Moreover, we found that the diversity and composition of the gut microbiome were stable after prolonged storage in eNAT at ambient temperature. These results suggest that eNAT is a suitable medium for fecal microbiome studies and may be particularly well suited for studies that include the home collection of fecal samples or are conducted in areas where cold chain storage is unavailable.

A variety of media has been used in the collection of fecal samples in studies of the gut microbiome. In this study, we compared samples processed in eNAT to samples processed in RNAlater solution, which is among the most frequently used preservation methods in microbiome studies (Choo et al., 2015). RNAlater is a storage reagent that stabilizes bacterial RNA and is particularly useful for bacterial gene expression profiling. However, the storage of fecal samples in RNAlater solution at ambient temperature results in substantial shifts in gut microbiome composition (Flores et al., 2015; Hale, Tan, Knight, & Amato, 2015; Kawada, Naito, Andoh, Ozeki, & Inoue, 2019). The advantages and disadvantages of two common stabilization media, Tris‐EDTA, and ethanol‐based solutions were previously discussed. Also, several other commercially available products were recently developed that also appear to be suitable for the preservation of DNA for gut microbiome analyses. OMNIgene^®^·Gut DNA stabilization kits (DNA Genotek) preserve fecal samples stored at ambient temperature for up to 60 days (Choo et al., 2015; Song et al., 2016). Similarly, long‐term storage of microbial DNA has been achieved with Whatman FTA^®^ matrix cards, which contain protein denaturants and buffers that lyze microbial cells and stabilize DNA (Rajendram et al., 2006).

Our results indicate that eNAT has several properties that make it a suitable medium for gut microbiome studies. First, eNAT contains compounds that rapidly and completely inactivate microorganisms, facilitating the safe handling of these samples by study participants and research personnel. Also, eNAT has a higher flash point than solutions containing high concentrations of ethanol and can be shipped without specific safety or temperature considerations. Finally, gut microbiome diversity and composition are preserved in fecal samples stored in eNAT at ambient temperature for 30 days. This suggests that eNAT may be particularly useful in settings in which refrigeration or cold chain storage is not available. In particular, eNAT could facilitate research in settings with limited infrastructure in which the collection and shipment of fecal samples for microbiome analyses might otherwise be cost‐prohibitive. Moreover, eNAT could improve participation in research that involves the home collection of fecal samples because the storage of fecal samples in the refrigerator may be unacceptable to some study participants.

This study has several limitations. First, the sample size was relatively small, which precluded us from evaluating for differential abundances of rare bacterial genera by the sample preparation method. Besides, these analyses used fecal samples from a cohort of children and adolescents undergoing hematopoietic stem cell transplantation. This enabled us to evaluate for bias in the ability of eNAT to preserve microbial communities with widely varied diversity and composition, but the gut microbiomes of patients included in this study likely differ from those in healthy adult populations (Kelly et al., 2019). All fecal samples were initially collected into 50‐mL tubes containing a small amount (~2.5–5.0 ml) of RNAlater solution, and it is possible that some RNAlater remained in the stool slurries that were transferred to the cryovials containing eNAT. This study focused only on fecal samples, and the findings cannot be generalized to samples collected from other ecological niches. Finally, although RNAlater solution is one of the most widely used media for microbiome studies, future work should compare samples stored in eNAT to samples stored in other stabilization media or fresh fecal samples.

In conclusion, we provide the first controlled experiment to assess the potential utility of eNAT as a stabilization media for fecal samples to be used in microbiome analysis. Our results suggest that eNAT may be a useful medium for studies of the gut microbiome. The ability of eNAT and other similar media to stabilize fecal samples at ambient temperature has the potential to improve the feasibility of conducting microbiome studies in settings without cold chain storage.

## AUTHORS' CONTRIBUTIONS

Rebecca Young: Conceptualization, Methodology, Software, Formal Analysis, Writing‐Original Draft, Visualization. Kirsten Jenkins: Investigation, Resources, Writing‐ Review & Editing. Felix Araujo‐Perez: Methodology, Software, Investigation. Patrick Seed: Conceptualization, Resources, Supervision, Writing ‐ Review & Editing. Matthew Kelly: Conceptualization, Writing ‐ Original Draft, Writing ‐ Review & Editing, Supervision, Funding Acquisition.

## CONFLICT OF INTEREST

eNAT media used in this experiment were provided by Copan Italia.

## ETHICS STATEMENT

Written informed consent was obtained from a legal guardian by study staff after a detailed explanation of the study procedures and the rights and protections of research participants. The study protocol was approved by the Duke University Institutional Review Board.

## Data Availability

The dataset supporting the conclusions of this study is available in the Sequence Read Archive (https://www.ncbi.nlm.nih.gov/bioproject/PRJNA578756). The statistical code used for data analyses has also been made publicly available (https://github.com/rebeccatree/eNAT).

## Appendix 1

**Table A2:** Median differences in the relative abundances of the 20 most highly abundant genera by sample preparation method

Genus	eNAT (‐80°C) – RNAlater (‐80°C)		eNAT (‐80°C) – eNAT (20°C)	
	Median difference in relative abundance	*p* ^a^	Median difference in relative abundance	*P* ^a^
*Bacteroides*	‐0.89%	.27	‐1.38%	.20
*Enterococcus*	‐1.75%	.89	‐1.62%	.25
*Parabacteroides*	0.22%	.09	‐0.96%	.06
*Clostridium* (Clostridiaceae)	0.15%	.06	2.07%	.04
*Faecalibacterium*	0.22%	.89	‐0.99%	.56
*Lactobacillus*	0.07%	.35	0.37%	.45
*Prevotella*	‐1.59%	.38	‐1.31%	.64
*Oscillospira*	0.37%	.29	‐0.10%	.23
*Ruminococcus* (Lachnospiraceae)	‐0.30%	.39	0.48%	.89
*Bifidobacterium*	‐0.10%	.13	0.69%	.55
*Streptococcus*	0.16%	.16	0.84%	.60
*Blautia*	0.24%	.38	‐0.03%	.25
*Ruminococcus* (Ruminococcaceae)	‐0.07%	.50	‐0.28%	.49
*Alistipes*	‐0.11%	.17	‐0.50%	.75
*Lachnospiraceae*	0.07%	.51	0.48%	.50
*Sutterella*	‐0.28%	.61	‐0.34%	.64
*Campylobacter*	0.68%	.99	1.01%	.99
*Clostridium* (Erysipelotrichaceae)	1.52%	.08	0.13%	.70
*Roseburia*	0.03%	.99	‐0.08%	.88
*Pediococcus*	0.07%	.50	‐0.02%	.68
